# Smartphone-Measured Sleep Health Risk Factors Detect Suicidal Thoughts and Behaviors Among Adolescents

**DOI:** 10.1016/j.jaacop.2026.06.001

**Published:** 2026-06-08

**Authors:** Jaclyn S. Kirshenbaum, David Pagliaccio, Esha Trivedi, Hanga Galfalvy, Karla J. Joyce, Katherine Durham, Giovanna Porta, Paul A. Bloom, Trinity C. Tse, Ashley Blanchard, Lauren S. Chernick, Ryann N. Crowley, Peter S. Dayan, Lauren E. Kahn, Jamie Zelazny, David A. Brent, Nicholas B. Allen, Randy P. Auerbach

**Affiliations:** a Columbia University, New York, New York; b New York State Psychiatric Institute, New York, New York; c University of Pittsburgh Medical Center, Pittsburgh, Pennsylvania; d Western Psychiatric Hospital, University of Pittsburgh Medical Center, Pittsburgh, Pennsylvania; e Columbia University Irving Medical Center, New York, New York; f University of Oregon, Eugene, Oregon

**Keywords:** youth, suicidal ideation, suicidal events, mobile sensors, experience sampling methodology

## Abstract

**Objective:**

Suicide deaths and poor sleep health in adolescents have significantly increased. Smartphone sensors (eg, accelerometry) provide an unobtrusive and scalable way to monitor sleep health over extended time periods. We hypothesized that poor weekly sleep health (ie, shorter and variable time in bed; late and variable bedtime) assessed through smartphone sensors would associate with weekly suicidal thoughts and behaviors (STB).

**Method:**

High-risk adolescents (N = 145, 13-18 years of age, 112 female) oversampled for STB (n = 114) installed the Effortless Assessment Research System application on their smartphones, allowing access to motion data over 6 months. Simultaneously, the app measured weekly suicidal ideation. Clinical interviews measured suicidal events (ie, attempts, specifc psychiatric hospitalizations, and emergency department events related to suicide risk) at 1-, 3-, and 6-month follow-up assessments. Mixed-effects logistic regression models tested whether the weekly average and variability of time in bed and bedtime related to next-week STB.

**Results:**

A 1-SD in weekly time in bed, relative to one’s typical weekly time in bed, related to a near 2-fold greater odds of a next-week suicidal event (odds ratio [OR] = 1.85, 95% CI = 1.01, 3.28, *p* = .045). This was driven by time in bed on weekdays (OR = 2.39, 95% CI = 1.19, 4.44, *p* = .013). Greater variability of bedtimes (ie, lower bedtime consistency) within a weekend, relative to one’s own typical weekend bedtime variability, predicted next-week suicidal ideation (OR = 1.91, 95% CI = 1.28, 2.69, *p* = .001).

**Conclusion:**

Results reveal proximal associations between smartphone-derived sleep measures and STB, highlighting the possibility of developing just-in-time interventions, focusing on which sleep behaviors to modify and when to intervene to reduce suicide risk.

## Plain language summary

Suicide deaths have significantly increased, and sleep health has worsened in adolescents over the past decade. In 145 adolescents, the authors measured passive sleep health behaviors via a smartphone app, sent weekly surveys, and conducted clinical interviews to assess suicide risk. Longer time in bed and greater variability in bedtimes related to a greater chance of next-week suicide risk. Results highlight potentially modifiable sleep health behaviors and allow for timely intervention to reduce suicide risk.

Despite considerable global prevention efforts, suicide remains a leading cause of death among adolescents.^1^ Suicide risk often changes rapidly and dynamically over short periods of time,^2^ which makes identification of proximal risk factors difficult but critical. Sleep health has long been recognized as an important indicator of suicidal thoughts and behaviors (STB),^3^, ^4^, ^5^, ^6^, ^7^ but there are few investigations into short-term links.^6^^,^^8^ This is especially relevant, as sleep health in adolescents has worsened over the past 10 years.^9^ Recent advances in smartphone technology allow unique access to probe dynamic changes in behavior, which may improve the short-term prediction of STB. Given that 95% of adolescents own a smartphone in the United States,^10^ there has been increased attention on real-time monitoring with smartphone sensors to passively track human behavior (eg, location, language) to predict risk states.^11^, ^12^, ^13^ Given evidence shows that smartphones can detect objective indicators of sleep health,^14^^,^^15^ this tool may be leveraged to signal suicidal risk over short periods of time.

Sleep health behaviors (eg, early and consistent bedtimes, adequate duration)^16^ undergo substantial changes during adolescence, including considerable shifts in weekend–weekday sleep behaviors.^17^ These changes can contribute to poor sleep health, which can impair psychological functioning (eg, emotion regulation)^18^ and thereby increase suicidal ideation risk.^6^ Retrospective assessments of past-week or past-month sleep disturbances^19^ show clear links to suicidal ideation in adolescents.^3^ Although perceived sleep quality may be reliably obtained from cross-sectional self-reports, other sleep behaviors (eg, duration, onset) can be particularly challenging to recall, given that individuals move<<? in and out of wakefulness throughout a typical sleep cycle.

To improve temporal resolution, researchers have used methods such as daily measurements of sleep^4^ and wearable accelerometers.^6^ However, most of these methods are not able to feasibly capture sleep measurments over long periods of time becaue of the limited battery life of wearables and low daily diary participant compliance.^20^ This is crucial, as critical transition periods when suicide risk is greatest (eg, 90 days post hospitalization)^21^ often persist for several months. In addition, sleep varies on a night-to-night basis, and recent work has pointed to sleep inconsistency, or intraindividual variability (ie, daily variation around the mean) as another important, yet underexplored factor that is associated with poor outcomes, including negative mood.^22^ As adolescents are vulnerable to sleep inconsistency across weekdays and weekends, it is important to use methods that may be able to capture temporally sensitive effects, which require monitoring over extended time frames. Moreover, improving scalable sleep research is important to reach a wider net of at-risk adolescents, particularly those living in under-resourced areas who experience sleep health disparities^23^ and may benefit from more accessible clinical tools.

For many youth, their smartphones are the last thing that they use before bed and the first thing that they pick up in the morning (including turning off an alarm).^24^ Accordingly, smartphone sensors (ie, accelerometry) may provide an unobtrusive and scalable way to monitor daily sleep health (bedtimes, duration) in real time over longer time periods. Research^15^^,^^25^, ^26^, ^27^ shows that smartphone sensor-derived sleep metrics are concordant with self-reported sleep and actigraphy, particularly among young people. In a separate sample using the Effortless Assessment Research Systems (EARS) application,^28^ the same app used in the current study, day-to-day sleep features (ie, bedtime, risetime, duration of time in bed) were significantly correlated among daily diary, actigraphy, and EARS.^15^ Collectively, early evidence shows that smartphone-based sleep measurement is aligned with established methods and may provide a feasible alternative to measuring day-to-day sleep patterns in a scalable way. As a whole, sleep health is an important indicator of STB, and smartphones may be able to detect real-time objective indicators of sleep health that may enable scalable detection of proximal suicidal risk over extended time frames.

This analysis examined smartphone sleep metrics in combination with weekly experience sampling methodology (ESM) and clinical interviews to predict risk for short-term suicidal ideation and suicidal events among adolescents. Analyses focused on bedtimes and time in bed as 2 aspects of sleep health that are profoundly affcted during adolescenc and are linked with suicidal ideation.^6^^,^^29^ We hypothesized that diminished sleep health (ie, late and more inconsistent bedtimes; short and more inconsistent duration) would prospectively associate with weekly suicidal ideation and suicidal events (attempts, hospitalizations, emergency department visits for suicide-related concerns).

## Method

### Participants

Adolescents (13-18 years of age) were recruited from the greater New York, New York and Pittsburgh, Pennsylvania areas to participate in a longitudinal study^11^^,^^30^, ^31^, ^32^ (Mobile Assessment for the Prediction of Suicide [MAPS]). Data were collected between November 2018 and April 2023, with participants recruited through psychiatric outpatient programs, pediatric emergency departments (EDs), medical center research registries, and social media. Adolescent assent and legal guardian consent for youth to participate were obtained for participants younger than 18 years of age. Adolescents 18 years of age completed informed consent. Eligible participants (N = 223) were fluent in English (guardians could provide permission in English or Spanish), 13-18 years of age, owned a personal smartphone (Android, iPhone 7, or later model), and reported a current depressive, anxiety, and/or substance use disorder, which was assessed via diagnostic interviews (Mini International Neuropsychiatric Interview for Children and Adolescents [MINI-KID] Version 7.02.20).^33^ The study was enriched for STB (n = 64 reporting a past-year suicide attempt and current ideation; n = 93 reporting current suicidal ideation but no history of attempt; n = 66 reporting no ideation or history of attempt [psychiatric controls], which was based on the baseline Scale for Suicide Ideation^34^ ≥4, a self-reported measure, and the Self-Injurious Thoughts and Behaviors Interview.^35^ Participants were excluded if they reported an imminent risk of suicide, active mania, lifetime psychosis, or an autism spectrum diagnosis. Participants consented to the use of de-identified data in published reports. The New York State Psychiatric Institute Institutional Review Board (IRB) approved study procedures (Protocol #7630) and was the IRB of record for both study sites.

At baseline, participants completed self-report measures assessing demographic information and psychiatric symptoms. They also installed the Effortless Assessment Research Systems (EARS) application^28^ on their smartphones, which delivered weekly STB surveys, daily mood surveys, and collected passive sensor data. Follow-up self-report and clinical assessments were administered during the 1-month, 3-month, and 6-month follow-up assessments.

### EARS Sleep Data

The EARS app uses built-in smartphone sensors to passively collect motion data and to estimate bedtimes (ie, going to bed) and risetimes (ie, waking up). The EARS app implemented motion state detection APIs in May 2020 for iOS and in June 2020 for Android, which uses the phone’s accelerometer, gyroscope, keyboard, and other available sensors to detect various states of motion (stationary, walking, running etc). These API-detected states are more precise for deriving sleep than are accelerometer data alone. EARS uses the CMMotionActivity API (iOS) and Activity Recognition Transition API (Android) to continuously detect changes in activity states. The start and end times of the longest stationary period in a given 24-hour day are used to isolate bedtimes (ie, going to bed) and risetimes (ie, waking up time), respectively. This longest period between bedtimes and risetimes operationalized time in bed. Android and iOS devices sample accelerometry data at different rates (10 Hz for Android; 50 Hz for iOS); thus, data were harmonized by down-sampling to 1 Hz.

Given that motion API detection was implemented part way through the study, motion data were collected in 153 participants for a total of 18,557 total days of motion data. We conducted a series of steps to remove observations that were likely misclassified by the API (this could be due to a variety of factors, including data missingness or leaving the phone outside the bedroom). Unlikely duration of time in bed (≤2 hours or ≥15 hours), bedtimes between 7 am and 7 pm, and rise times between 3 pm and 3 am were discarded (Figure S1, available online). After cleaning, 15,600 days (84%) of motion data were retained across 150 participants. We created weekly aggregates of the daily motion data (Wednesday to Tuesday) to align with ESM surveys, which were delivered every Wednesday (distributions in Figure S2, available online). We removed weeks with only 1 day of usable motion data (169 observations; 5 participants), which yielded 145 participants (n = 69 reporting a past-year suicide attempt and current ideation; n = 45 reporting current suicidal ideation but no history of attempt).

### ESM Smartphone Surveys

#### STB

Each Wednesday at 9 am, participants were asked, “In the past week, how often have you thought of killing yourself?” with ordinal response choices (1 = never, 2 = rarely, 3 = sometimes, 4 = often, 5 = all of the time), and “In the past week, did you make an attempt to kill yourself?” with binary response choices (yes/no). The presence of clinically significant suicidal ideation was binarized based on a response of 4 or higher. Clinically significant suicidal ideation (≥4) and the presence of an an attempt triggered our safety protocol for clinical staff to contact the participant to assess safety. When necessary, staff would bridge participants to emergency clinical services.^11^ The 9 am survey delivery time allowed participants sufficient time to respond (until 5 pm). This 8-hour period also allowed clinical staff ample time to respond within the same day if our suicide risk management plan was triggered.

#### Daily Mood

At 8 am each morning, participants were prompted with the question, “In general, how have you been feeling over the past day?” with continuous response choices ranging from 0 (very negative) to 100 (very positive). Daily mood responses were aggregated into weekly averages (Wednesday to Tuesday).

### Clinical Interviews

At baseline, trained study staff (bachelor’s degree level or higher, with ∼50 hours of interview training and direct oversight by a licensed clinical psychologist; interrater reliability *k* = 0.73) administered MINI-KID Version 7.02.20.^33^ The MINI-KID is a structured diagnostic interview that assesses current and prior *DSM-5* psychiatric disorders in youth. In addition, at the baseline, 1-, 3-, and 6-month assessments, participants were administered the Self-Injurious Thoughts and Behaviors Interview (SITBI)^35^ to assess the presence, frequency, and severity of suicidal thoughts and behaviors (STB; eg, ideation, plans, and actual, interrupted, or aborted attempts) and non-suicidal self-injury. At baseline, participants reported on lifetime STB history, whereas follow-up assessments focused on STB since the last assessment. All study staff received ∼50 hours of training prior to conducting clinical interviews. Supervised training included didactics, role playing, and direct observation, and weekly case conferences throughout the study.^11^ Staff conducted interviews only after receiving certification, and there were annual re-certifications to maintain clinical fidelity. Interrater reliability (IRR) was based on a random selection of 10% of baseline interviews by independent raters. The IRR on the baseline SITBI was excellent (*k* = 0.86). The IRR for follow-up SITBI assessments was not conducted because the utility of the SITBI for follow-up assessments was aimed at gathering specific information (ie, dates) regarding the occurrence of suicide attempts. Thus, there was little room for disagreement. Participants also completed a Service Use Interview at each assessment asking about psychiatric service use. The Columbia–Suicide Severity Rating Scale (C-SSRS)^36^ was used to assess imminent STB risk based on responses to the ESM smartphone suicidal ideation surveys or clinical interviews. We used the SITBI, Service Use Interview, C-SSRS, and weekly suicide event item to capture the date and presence of suicidal events (ie, attempts, psychiatric hospitalizations, and emergency department events related to suicide risk). All responses were based on the adolescent’s report. The presence of suicidal events each week was binarized for subsequent analyses.

### Self-Report Measures

#### Morningness–Eveningness Questionnaire

The Morningness–Eveningness Questionnaire (MEQ)^37^ is a 19-item questionnaire that assesses one’s preference for functioning in the morning or evening. Scores range from 16 to 86, with higher scores indicating a greater preference for morning. The MEQ in this sample had good internal consistency (α = .77).

#### Pittsburgh Sleep Quality Index

The Pittsburgh Sleep Quality Index (PSQI)^38^ is a self-reported questionnaire that probes individuals’ typical bedtime, risetime, hours of “actual sleep,” any trouble sleeping, and sleep quality over the last month. There are 7 components, or subscales, that, when combined, yield a total “sleep quality” score ranging from 0 to 21. Higher scores indicate worse sleep quality. The PSQI demonstrated an internal consistency of α = .67.

#### Beck Scale for Suicide Ideation

The Beck Scale for Suicide Ideation (SSI)^39^ is a 21-item self-reported scale for determining severity of suicidal ideation in the past 2 weeks. All participants completed the first 5 items, which evaluate the presence of suicidal ideation. Participants who endorse suicidal ideation complete the next 14 items, which measure the intensity of ideation, methods, capability, and preparation for an attempt. For the current analysis, we examined the first 5 items completed by all participants. The SSI demonstrated good internal consistency (α = .84).

#### Mood and Feelings Questionnaire

The Mood and Feelings Questionnaire (MFQ; Short Version)^40^ asks about individuals’ depressive symptoms without asking about STB. Scores range from 0 to 26, with higher scores indicating worse depressive severity. The MFQ showed good internal consistency (α = .88).

#### Snaith–Hamilton Pleasure Scale

The Snaith–Hamilton Pleasure Scale (SHAPS)^41^ assesses one’s ability to experience pleasure. Scores range from 14 to 56, with higher scores demonstrating a greater ability to experience pleasure. Scores were reverse coded, so that higher scores indicated greater anhedonia. The SHAPS demonstrated good internal consistency (α = .88).

#### Screen for Anxiety Related Emotional Disorders

The Screen for Anxiety Related Emotional Disorders (SCARED; Short Version)^42^ screens for the presence of anxiety disorders in children. Scores range from 0 to 10, with higher scores indicating higher anxiety severity. The SCARED showed excellent internal consistency (α = .93).

### Data Analytic Approach

#### Covariates for Analyses

To avoid model overfitting, variables were included as covariates in subsequent models based on whether they were significantly associated with weekly suicidal events or clinically significant suicidal ideation. We tested whether certain time-varying covariates (ie, summer break, COVID-related school closures, weekly mood, and weeks since baseline) were associated with weekly suicidal events and suicidal ideation. Supplement 1, available online, provides analyses regarding COVID-related school closures. In addition, although we focus on week-to-week associations between smartphone-sensor sleep metrics and STB, we tested whether baseline time-invariant demographic and symptom severity variables were associated with our outcome variables, to understand the unique variance explained by weekly sleep estimates adjusting for between-person participant characteristics that have been consistently associated with STB in the literature (eg, sex, depression). The following variables were tested: age at baseline, sex (female vs male), phone type (iPhone vs Android), baseline suicidal ideation (SSI), baseline depression severity (MFQ), baseline circadian preference (MEQ), baseline anxiety (SCARED), baseline anhedonia (SHAPS), and baseline sleep quality (PSQI).

#### Weekly Sleep Health and Suicidal Risk

Analyses were conducted in R version 4.4.2. Weekly aggregates of sleep health features included the mean and SD within the week (Wednesday to Tuesday) leading up to each weekly suicidal ideation prompt or suicidal event. Separate logistic mixed-effects models (*glmer* function) were conducted to examine associations between weekly time in bed and bedtime in relation to next-week suicidal events and suicidal ideation, respectively (ie, time lagged).

All models included a random intercept per participant. All sleep variables were person-mean centered. Accordingly, estimates for the weekly mean and SD of sleep metrics are interpreted as estimates relative to the person mean (ie, within-participant). Correlations between person-centered means and SDs were small (0.03 ≤ *r ≤* 0.19*, ps ≤* .323); therefore, means and SDs for each sleep metric were included in the same model.

### Sensitivity Analyses

Given expected weekday/weekend differences in sleep, all models were repeated to separate weekday and weekend sleep in relation to STB. Code for data analyses can be found here.

## Results

#### Participant Characteristics

Participant characteristics for the sample with usable motion data (N = 145) are summarized in Table 1, and differences in characteristics among risk groups are summarized in Table S1, available online. Eleven participants experienced 18 suicidal events during the motion collection period. There were 9 aborted attempts, 3 suicide attempts, 3 emergency department visits, and 3 hospitalizations. For ESM surveys, participants completed approximately 38% of weekly suicide risk surveys over the course of 25 weeks. We have previously reported that STB and psychiatric symptoms are not associated with missing data in the larger sample.^31^ However, we conducted analyses in this specific sample to examine whether weekly survey compliance is associated with STB or related symptoms. Cross-sectional analyses showed that the proportion of missing weekly surveys is not different between participants with suicide events and those without suicide events *t*(12.26) = 0.18, *p* = .861. The proportion of missing weekly surveys is also not associated with time-varying suicide events (OR = 0.72, 95% CI = 0.19-2.79, *p* = .639) or the likelihood of endorsing weekly suicidal ideation (OR = 0.62, 95% CI = 0.38-1.02, *p* = .062). In addition, the proportion of missing surveys is not associated with poorer baseline mental health, including suicidal ideation (β = −0.05, 95% CI = −0.22 to 0.12, *p* = .545), anhedonia (β = −0.06, 95% CI = −0.22 to 0.11, *p* = .519), and depression (β = −0.09, 95% CI = −0.252 to 0.08, *p* = .322). Throughout the study period, there were 66 instances of clinically significant suicidal ideation across 33 (23%) participants.

**Table 1:** Participant Characteristics (N = 145)

Variable	Mean (SD) or n (%)
Age, y	16.41 (1.71)
Sex, female	112 (77.24%)
Gender, female	107 (73.79%)
Race	—
American Indian/Alaska Native	1 (0.69%)
Asian	16 (11.03%)
Black	23 (15.86%)
Multiracial	16 (11.03%)
Unknown	9 (6.21%)
White	80 (55.17%)
Hispanic	27 (18.62%)
Site	
CUIMC	71 (48.97%)
PITT	74 (51.03%)
Income	—
<25 k	8 (7.34%)
25-100 k	51 (46.79%)
>100 k	50 (45.87%)
iPhone (iOS)	126 (86.9%)
Risk group	—
Psychiatric condition without ideation/attempt	31 (21.38%)
Current ideation	45 (31.03%)
History of attempt	69 (47.59%)
Psychiatric medication throughout study	115 (79.31%)
Change in psychiatric medication	24 (16.55%)
EARS sleep features, person-level	
Time in bed, h	7.58 (1.16)
Bedtime	12:57 am (1:23 am)

Note: Psychiatric condition without ideation/attempt includes current depressive, anxiety, and/or substance use disorder. CUIMC = Columbia University Irving Medical Center; EARS = Ecological Assessment of Research Systems; PITT = University of Pittsburgh.

#### Covariates

In the models testing potential covariates, weeks since baseline was the only variable associated with weekly suicidal events (OR = 0.52, 95% CI = 0.29-0.96, *p* = .036). Therefore, the number of weeks since baseline was included as a covariate in models predicting events. Suicidal ideation severity at baseline was the only variable uniquely associated with weekly clinically significant suicidal ideation (OR = 2.55, 95% CI = 1.50-4.34, *p* = .001). Therefore, only baseline suicidal ideation was included as a covariate in subsequent models testing the association between weekly sleep features and weekly clinically significant suicidal ideation.

#### Sleep Features Predicting Next-Week Suicidal Events

Of the 145 participants with usable motion data, 128 participants had sufficient motion across at least 2 weeks of the study; 127 participants had at least 2 weeks of weekday data, and 119 participants had at least 2 weeks of weekend data. A 1-SD increase in weekly time in bed, relative to one’s typical weekly time in bed, related to a nearly 2-fold greater odds of a next-week suicidal event (OR = 1.85, 95% CI = 1.01-3.28, *p* = .045). This was driven by weekday (ie, school week) time in bed (OR = 2.39, 95% CI = 1.19-4.44, *p* = .013) (Table 2, Figure 1). Examining this model in hours revealed that every hour in increased average time in bed during weekdays related to a 1.98 greater odds (95% CI = 1.08-3.97, *p* = .028) of a next-week suicidal event. To address whether participants might already be in a prodromal phase of suicide behavior, we tested whether time in bed 2 weeks before increased the likelihood of a suicide event. Results were in the same direction 2 weeks before, but the model was not statistically significant (OR = 1.48, 95% CI = 0.88-2.42, *p* = .138). No other sleep features were associated with next-week suicidal events (*ps* ≥ .129) (Table 2; Table S2, available online). Participants varied in regard to the number of suicidal events that they experienced. We conducted supplemental analyses for weekday time in bed in relation to sucide events regarding the number of events experienced per participant (Supplement 1: Sensitivity Analysis 1.1, available online). We also assessed model robustness by bootstrapping a Bayesian mixed model and by performing univariate analyses, both of which yielded estimates similar to our original solution (Supplement 1: Sensitivity Analysis 1.2, Figure S3, available online).

**Table 2:** Associations Between Time in Bed and Suicidal Events

Predictors	Next-week SE			Next-week SE			Next-week SE		
	aOR	95% CI	*p*	aOR	95% CI	*p*	aOR	95% CI	*p*
Whole-week TIB SD (pmean)	0.63	0.36-1.14	.130						
Whole-week TIB mean (pmean)	**1.85**	**1.01-3.28**	**.045**						
Weeks since baseline	**0.53**	**0.29-0.98**	**.045**	**0.52**	**0.28-0.98**	**.042**	**0.45**	**0.21-0.96**	**.040**
Weekday TIB mean (pmean)				**2.39**	**1.19-4.46**	**.013**			
Weekday TIB SD (pmean)				0.76	0.42-1.39	.380			
Weekend TIB mean (pmean)							0.82	0.42-1.65	.599
Weekend TIB SD (pmean)							0.67	0.33-1.38	.284
Random effects									
σ^2^	3.29			3.29			3.29		
τ_00_	35.97 _ID_			39.02 _ID_			40.44 _ID_		
ICC	0.92			0.92			0.93		
n	128 _ID_			127 _ID_			119 _ID_		
Observations	2,736			2,664			1,995		
Marginal *R*^2^ / conditional *R*^2^	0.022 / 0.918			0.025 / 0.924			0.019 / 0.926		

Note: aOR = adjusted odds ratio; ICC = intraclass correlation coefficient; pmean = person-centered mean; TIB = time in bed.

**Figure 1:**
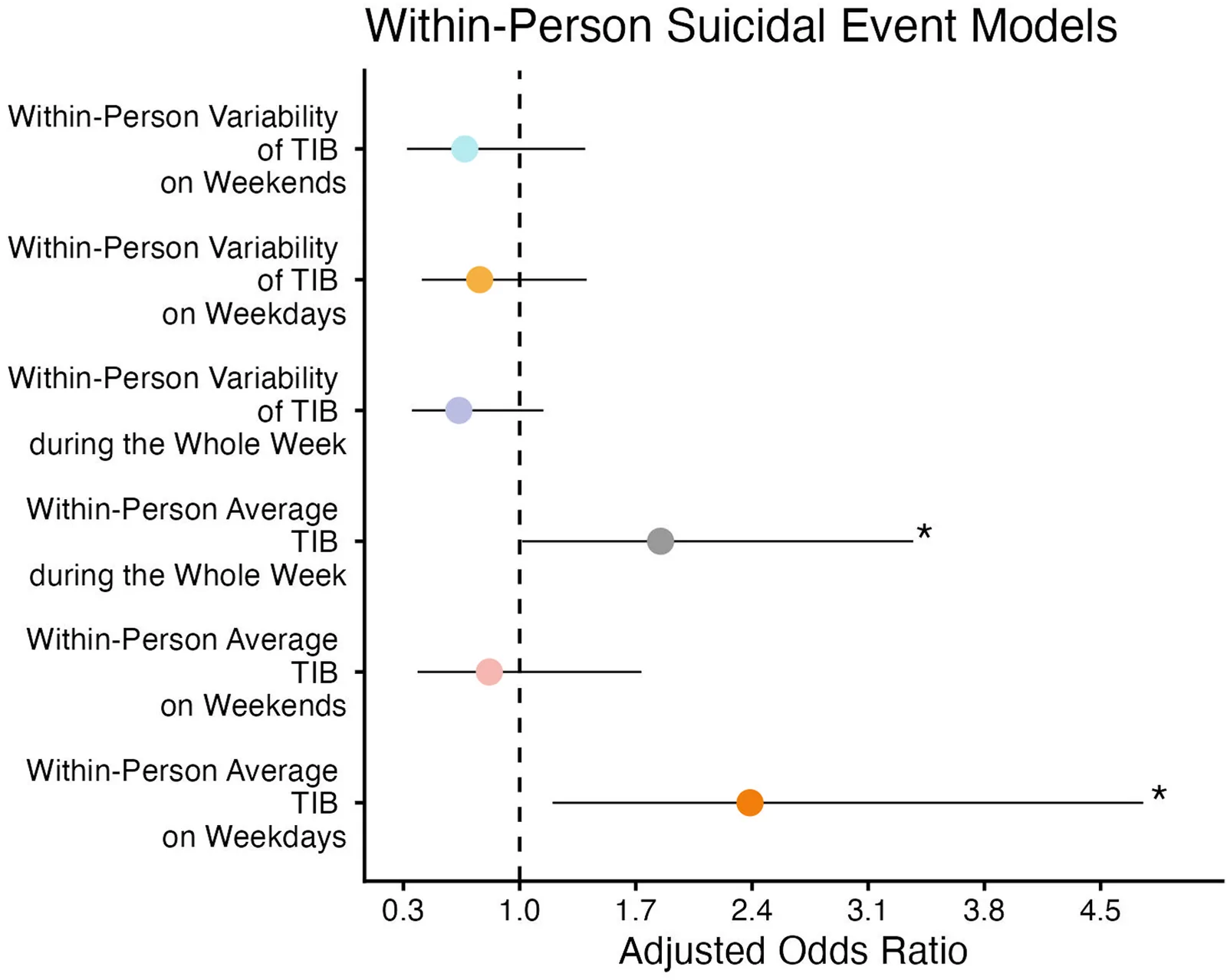
Suicide Events Models ***Note:****TIB = time in bed.*

#### Sleep Features Predicting Next-Week Clinically Significant Suicidal Ideation

Of the 145 participants with usable motion data, 115 participants had sufficient motion with at least 2 weeks of survey data; 114 participants had at least 2 weeks of weekday survey data; and 102 participants had at least 2 weeks of weekend survey data. There were no significant associations between whole-week time in bed or bedtime in relation to next-week clinically significant suicidal ideation (0.75 ≤ OR ≤ 1.26, *p*s ≥ .187) (Table 3; Table S3, available online). However, sensitivity analyses revealed that a 1-SD increase in weekend variability of bedtime (ie, higher bedtime inconsistency), relative to one’s typical weekend bedtime variability, related to a 1.91 greater odds of next-week clinically significant suicidal ideation (95% CI = 1.28-2.69, *p* = .001) (Table 3, Figure 2A, B). Examining this same model in hours reveals that every hour in increased bedtime variability on weekends relative to one’s typical weekend bedtime variability related to a 2.08 greater odds (95% CI = 1.35-2.80, *p* < .001) of next-week clinically significant suicidal ideation. We also performed analyses at a lower threshold (≥3), which yielded similar results (OR = 1.29, 95% CI = 1.03-1.59, *p* = .029). Given that the binary classification may have drawbacks in grouping participants with “no ideation” and “infrequent” ideation in the same category, we re-analyzed these data on the original continuous responses of suicidal ideation, which yielded similar results (β = 0.06, 95% CI = 0.02-0.10, *p* = .003).

**Table 3:** Associations Between Bedtime and Clinically Significant Suicidal Ideation

Predictors	Next-week SI			Next-week SI			Next-week SI		
	aOR	95% CI	*p*	aOR	95% CI	*p*	aOR	95% CI	*p*
Intercept	0.01	0.01-0.02	**<.001**	0.01	0.01-0.03	**<.001**	0.01	0.00-0.02	**<.001**
Whole-week bedtime SD (pmean)	1.15	0.84-1.55	.392						
Whole-week bedtime mean (pmean)	0.88	0.65-1.20	.422						
Baseline suicidal ideation severity	**3.03**	**1.92-4.73**	**<.001**	**3.00**	**1.90-4.63**	**<.001**	**3.71**	**1.99-6.68**	**<.001**
Weekday bedtime mean (pmean)				0.98	0.71-1.35	.913			
Weekday bedtime SD (pmean)				1.04	0.76-1.43	.799			
Weekend bedtime mean (pmean)							0.75	0.51-1.14	.187
Weekend bedtime SD (pmean)							**1.91**	**1.28-2.69**	**.001**
Random effects									
σ^2^	3.29			3.29			3.29		
τ_00_	2.56 _ID_			2.54 _ID_			4.63 _ID_		
ICC	0.44			0.44			0.58		
n	115 _ID_			114 _ID_			102 _ID_		
Observations	1,341			1,307			1,013		
Marginal *R*^2^ / conditional *R*^2^	0.175 / 0.537			0.169 / 0.531			0.207 / 0.671		

Note: aOR = adjusted odds ratio; ICC = intraclass correlation coefficient; pmean = person-centered mean.

**Figure 2:**
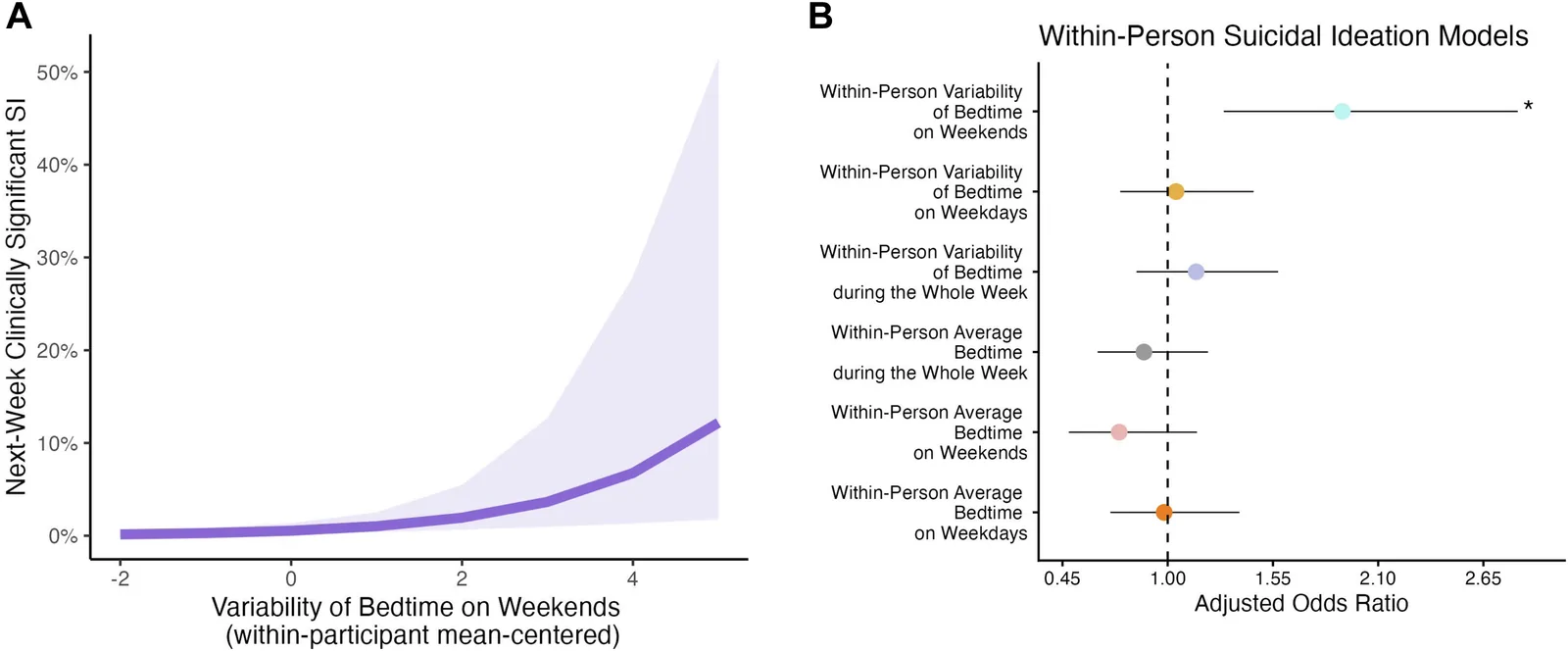
Suicide Ideation Models

## Discussion

Over the past decade, suicide deaths in adolescents have increased by ∼40%,^43^ which aligns with increasing trends of insufficient sleep in youth.^44^ Although several meta-analyses have highlighted the strong link between sleep disturbances and suicide risk,^45^^,^^46^ few studies have documented proximal links between sleep health and STB. Suicide risk within individuals varies over time, which makes continuous monitoring of contributing factors to STB critical in high-risk adolescents. Leveraging scalable tools to detect proximal increases in suicide risk over extended time periods may mitigate the sharp increases in suicide attempts and deaths. Poor sleep health has been long recognized as a risk factor for suicide. Advances in smartphone technology make it possible to use existing sensors to continuously monitor sleep behaviors, which afford unique access to temporally sensitive effects (eg, week to week, weekend/weekday effects) of sleep in relation to suicide risk. We aimed to test whether sleep patterns measured through smartphone sensors are related to weekly suicidal events and ideation over 6 months in high-risk adolescents. Overall, our findings showed that longer within-person time in bed during weekdays predicted next-week suicidal events and increased within-person variability of bedtimes (ie, higher bedtime inconsistency) on weekends predicted next-week increases in clinically significant suicidal ideation.

Longer than typical time in bed during the week was associated with an approximate 2-fold increase in suicidal events the following week. Upon separating weekend and weekday models, we found that this effect was detected only on weekdays. Although time in bed and sleep duration tend to be correlated,^47^ these constructs are distinct. For instance, prolonged sleep onset latency and more frequent wake periods after sleep onset could both result in longer time in bed and shorter sleep duration. Therefore, although this study found longer time in bed to be associated with suicide events, it is not surprising that prior research has shown that shorter sleep duration is associated with increased suicidal ideation in a similarly acute population monitored over 3 months.^6^ Given that our finding of longer than typical time in bed predicted next-week suicidal events but not suicidal ideation, it is also possible that multidimensional sleep health behaviors differentially explain the presence of proximal suicidal events and suicidal ideation. Longer than typical time in bed on a given school week could reflect the desire to sleep or rest more if sleep quality was low the week before. Because both hypersomnia and insomnia are common in depressed adolescents,^48^ longer time in bed during the school week could also reflect common depressive and anhedonic behaviors, such as reduced motivation to attend school. Absenteeism has been associated with suicidal behaviors,^49^ which could indicate a state of heightened anhedonia, a known risk factor for suicide.^50^ A recent daily diary study provided initial evidence for anhedonia as a mechanism explaining the association between sleep problems and next-day suicidal ideation in high-risk adolescents.^51^ Longer than typical time in bed could also reflect greater homestay or longer amounts of time spent at home. Recent research in the same sample showed that greater homestay is associated with proximal risk for suicidal events,^11^ which may also be associated with increased isolation, social withdrawal, and anhedonia. In sum, longer than typical time in bed in at-risk youth may indicate heightened anhedonia or another signal of distress that could provide insights into suicide risk.

Higher within-person bedtime inconsistency on weekends yielded a greater risk of clinically significant suicidal ideation the next week. This finding aligns with prior studies showing that intraindividual variability of bedtimes is consistently associated with lower well-being,^52^^,^^53^ and that greater bedtime consistency (ie, going to bed at the same time each night) promotes better sleep health and other mental health outcomes.^22^ Importantly, we were able to detect that inconsistency on weekends, but not weekdays, was significantly associated with proximal suicidal ideation. Prior ecological momentary assessment (EMA) studies in high-risk adolescents show that subjective sleep onset latency, nightmares, and pre-sleep rumination predicted next day suicidal ideation.^4^ These factors that make it difficult to fall asleep may lead to greater irregularity in bedtimes. Bedtime inconsistency could also be developmentally relevant, as bedtime routines become more autonomous throughout adolescence because of social influences,^54^ particularly on weekends. Understanding which environmental (eg, screen time)^55^ and internal (eg, rumination) factors contribute to more variable bedtimes on weekends may help to inform how to promote more consistent bedtime routines as a protective factor against suicidal ideation for high-risk adolescents.

This study had several limitations. First, smartphone sensors using the EARS app are currently unable to detect naps and other sleep health features (eg, sleep onset latency, wake after sleep onset). It is possible that these more nuanced sleep behaviors, as reported in prior studies (eg, Glennn *et al.*, Ratzon *et al.*, Koyawala *et al.*^4^^,^^7^^,^^56^), are associated with suicide risk as well. Sleep health behaviors, such as naps, fragmented sleep, and sleep onset latency would require a more sensitive instrument than smartphone sensors. Although the sleep metrics examined in this study—namely, time in bed and bedtime—have been validated with self-report and actigraphy,^14^^,^^47^^,^^57^, ^58^, ^59^ other sleep metrics (eg, onset latency, wake after sleep onset, sleep efficiency) measured with smartphones have not shown such alignment.^60^^,^^61^ Thus, it may be that smartphone sensors can reliably detect basic features of sleep health (ie, bedtime, risetime, time in bed) but may be limited in detecting other features that require increased sensitivity to subtle movements (eg, motionless scrolling). There are certain benefits and trade-offs that come with each method. In this case, the scalability, feasibility, and accessibility of smartphones to measure sleep over extended periods of time comes with a trade-off of the accuracy that we may otherwise get with research-grade devices (eg, actigraphy). The aim of this study was to test whether smartphone devices could be used to detect sleep health patterns that are proximally related to STB over a 6-month period, which can be challenging to measure using other established methods that rely on compliance. Second, there were relatively few suicidal events, which affects the ability to detect effects. Independent datasets are needed to test whether results can be reproduced. Third, the study was designed to capture weekly periods of clinically significant suicide risk assessed via weekly experience sampling. Accordingly, we used weekly aggregates of sleep health metrics and mood to identify factors that precede these risk states. The design, however, cannot capture variability that may occur within days, which also reflects an important issue related to youth suicide risk. Finally, this sample comprisd mostly female, White, and iPhone users, which limits the generalizability of our findings. To increase generalizability to more populations, future research may benefit from oversampling on these characteristics that were underrepresented in this sample. This is important, given that Black and Hispanic youth tend to show greater disruptions in sleep relative to their White counterparts,^62^ and minoritized youth tend to underreport STB.^63^ Given the scalability of using smartphone sensors, this method may in fact achieve goals to increase the presence of those individuals typically underrepresented in research (eg, Black and Hispanic youth), thereby increasing generalizability and reducing health disparities.

Although the effect sizes for time in bed and bedtime were small, this study shows that it is feasible to use smartphones to detect whether sleep health behaviors proximally relate to weekly suicidal ideation and events. There are several use cases for leveraging passive smartphone sensor data. One potential avenue could improve the detection of proximal risk in several contexts, which would allow for the timely delivery of additional clinical support. For instance, families and schools may be able to use these data in combination with other signs of distress (eg, greater homestay, heightened reactivity to negative interpersonal events,^6^ anhedonia^51^) to signal the need for careful monitoring. Future studies with larger samples may also be able to examine personalized profiles of disrupted sleep health and other signs of STB to predict different phases of risk. Alternatively, smartphones may serve as a useful harbinger of inflections in suicidal risk that can guide both the adolescent and their treatment team to target sleep health and to reduce imminent suicidal risk. For example, integrating passive sensor data with established therapies (eg, dialectical behavior therapy) may improve symptom reduction.^64^ In fact, recent research in depressed young adults shows that passive smartphone sensor data were able to guide behavioral activation, which reduced depressive symptoms over time.^65^^,^^66^ Taken together, measuring sleep via smartphone sensors may yield several actionable inroads in reducing STB.

Sleep health in adolescents has worsened over the past 10 years,^9^ which may be one factor contributing to the rising suicide rates in this age group. Ultimately, leveraging smartphones to measure sleep health behaviors in combination with other clinical factors may inform how and when to provide timely feedback to at-risk adolescents to prevent suicide risk.

## CRediT authorship contribution statement

**Jaclyn S. Kirshenbaum:** Writing – review & editing, Writing – original draft, Formal analysis, Data curation. **David Pagliaccio:** Writing – review & editing, Supervision, Investigation, Formal analysis, Data curation. **Esha Trivedi:** Writing – review & editing, Investigation, Formal analysis, Data curation. **Hanga Galfalvy:** Methodology, Formal analysis. **Karla J. Joyce:** Writing – review & editing, Project administration, Investigation, Data curation. **Katherine Durham:** Writing – review & editing, Supervision, Project administration, Investigation. **Giovanna Porta:** Writing – review & editing, Project administration, Investigation, Data curation. **Paul A. Bloom:** Writing – review & editing, Formal analysis, Data curation. **Trinity C. Tse:** Writing – review & editing, Project administration, Investigation, Data curation. **Ashley Blanchard:** Writing – review & editing, Resources, Investigation. **Lauren S. Chernick:** Writing – review & editing, Resources, Project administration, Investigation. **Ryann N. Crowley:** Software, Methodology, Data curation. **Peter S. Dayan:** Writing – review & editing, Supervision, Resources, Project administration. **Lauren E. Kahn:** Software, Methodology, Data curation. **Jamie Zelazny:** Resources, Project administration, Investigation. **David A. Brent:** Writing – review & editing, Supervision, Resources, Methodology, Investigation, Funding acquisition, Conceptualization. **Nicholas B. Allen:** Writing – review & editing, Supervision, Software, Methodology, Investigation, Funding acquisition, Conceptualization. **Randy P. Auerbach:** Writing – review & editing, Supervision, Resources, Methodology, Investigation, Funding acquisition, Formal analysis, Conceptualization.
