# Supplementary material for: Smartphone-Measured Sleep Health Risk Factors Detect Suicidal Thoughts and Behaviors Among Adolescents
Source: JAACAP Open. 2026 Jun 8;4(5):756–67. doi: 10.1016/j.jaacop.2026.06.001 (PMC13643448; doi:10.1016/j.jaacop.2026.06.001)
Supplement: Supplementary file 1 [file mmc1.docx]

**Smartphone Measured Sleep Health Risk Factors Detect**

**Suicidal Thoughts and Behaviors among Adolescents**

**Supplement 1**

**Supplementary Motion Analysis.**

Supplementary cleaning discarded “unlikely” bedtimes and risetimes. We defined bedtimes and risetimes in the following way: We categorized “true” bedtime and “true” rise time periods as “late”, “very late”, “unlikely”, “very early”, “early”, and “normal”.

**Figure S1.**

**Figure S2.** Distributions of weekly sleep health features.

**Analysis regarding COVID-related school closures, sleep health, and suicide risk.**

Research has found that emergency department visits for suicidal thoughts and behaviors (STB) in youth increased during the COVID-19 pandemic.^1^ Additionally, sleep patterns changed for some adolescents during the pandemic with the interruption to in-person learning and limited opportunities for in-person social interactions.^2^ Therefore, we tested whether COVID-related school closures during the data collection period was associated with clinically significant suicidal ideation or suicide events. We did not find evidence that this association existed beyond baseline suicidal ideation severity. We also tested whether COVID-related school closures during data collection period was associated with sleep health patterns. We did not find evidence of a significant association between COVID-related school closures and time-in-bed (p=.607) or bedtime (p=.946). Weekend and weekday sleep health patterns also were not significantly different as a function of the pandemic (ps≥.299).

| **Table S1. Differences among Risk Groups** | | | | | |
| --- | --- | --- | --- | --- | --- |
| **Variable** | **Control (N=31)** | **Attempter (N=45)** | **Ideator (N=69)** | **Stat** | **p** |
| Age ** | 17.03 (1.47) | 15.78 (1.78) | 16.54 (1.64) | F=5.69 | 0.004 |
| Sex (F) | 22 (70.97%) | 37 (82.22%) | 53 (76.81%) | X2=1.34 | 0.51 |
| Gender (F) | 22 (70.97%) | 34 (75.56%) | 51 (73.91%) | X2=3.10 | 0.54 |
| Race |  |  |  |  |  |
| American Indian/ Alaska Native | 0 (0%) | 0 (0%) | 1 (1.45%) |  |  |
| Asian | 4 (12.9%) | 3 (6.67%) | 9 (13.04%) |  |  |
| Black | 2 (6.45%) | 10 (22.22%) | 11 (15.94%) |  |  |
| Multiracial | 4 (12.9%) | 5 (11.11%) | 7 (10.14%) |  |  |
| Unknown | 2 (6.45%) | 5 (11.11%) | 2 (2.9%) |  |  |
| White | 19 (61.29%) | 22 (48.89%) | 39 (56.52%) |  |  |
| Hispanic | 5 (16.13%) | 8 (17.78%) | 14 (20.29%) | X2=0.27 | 0.87 |
| SITE |  |  |  | X2=0.78 | 0.68 |
| CUIMC | 13 (41.94%) | 23 (51.11%) | 35 (50.72%) |  |  |
| PITT | 18 (58.06%) | 22 (48.89%) | 34 (49.28%) |  |  |
| Income |  |  |  |  |  |
| <25k | 4 (15.38%) | 2 (6.25%) | 2 (3.92%) |  |  |
| 25-100k | 12 (46.15%) | 14 (43.75%) | 25 (49.02%) |  |  |
| >100k | 10 (38.46%) | 16 (50%) | 24 (47.06%) |  |  |
| iPhone (iOS) | 27 (87.1%) | 39 (86.67%) | 60 (86.96%) | X2=0.00 | 1 |
| TIB in hours (SD) | 7.51 (1.05) | 7.63 (1.29) | 7.57 (1.13) | F=0.10 | 0.9 |
| Average Bedtime (SD) | 1:17AM (1:29AM) | 12:38AM (1:20AM) | 1:01AM (1:21AM) | F=2.19 | 0.12 |
| *Note.* TIB=Time-in-Bed. Formal comparisons among race, site, and income could not be approximated due to low numbers within each risk group. ** p<0.01. | | | | | |

| **Table S2. Associations between Bedtime and Suicide Events** | | | | | | | | | |
| --- | --- | --- | --- | --- | --- | --- | --- | --- | --- |
|  | **Next-Week SE** | | | **Next-Week SE** | | | **Next-Week SE** | | |
| *Predictors* | *aOR* | *95% CI* | *p* | *aOR* | *95% CI* | *p* | *aOR* | *95% CI* | *p* |
| Whole-Week Bedtime SD (pmean) | 0.97 | 0.56 – 1.68 | 0.913 |  |  |  |  |  |  |
| Whole-Week Bedtime Mean (pmean) | 1.07 | 0.61 – 1.86 | 0.819 |  |  |  |  |  |  |
| Weeks Since Baseline | **0.52** | **0.28 – 0.96** | **0.036** | **0.51** | **0.28 – 0.96** | **0.036** | **0.41** | **0.19 – 0.90** | **0.025** |
| Weekday Bedtime Mean (pmean) |  |  |  | 0.89 | 0.50 – 1.62 | 0.719 |  |  |  |
| Weekday Bedtime SD (pmean) |  |  |  | 1.10 | 0.63 – 1.93 | 0.732 |  |  |  |
| Weekend Bedtime Mean (pmean) |  |  |  |  |  |  | 0.96 | 0.52 – 1.79 | 0.904 |
| Weekend Bedtime SD (pmean) |  |  |  |  |  |  | 1.47 | 0.81 – 2.60 | 0.207 |
| *Random Effects* |  |  |  |  |  |  |  |  |  |
| σ^2^ | 3.29 |  |  | 3.29 |  |  | 3.29 |  |  |
| τ_00_ | 34.42_ID_ | |  | 35.86 _ID_ | |  | 40.92 _ID_ | |  |
| ICC | 0.91 |  |  | 0.92 |  |  | 0.93 |  |  |
| N | 128 _ID_ |  |  | 127 _ID_ |  |  | 119_ID_ |  |  |
| Observations | 2736 |  |  | 2664 |  |  | 1995 |  |  |
| Marginal R^2^ / Conditional R^2^ | 0.012 / 0.914 |  |  | 0.011 / 0.917 |  |  | 0.021 / 0.927 |  |  |
| *Note.* SD = standard deviation; pmean = person-centered; aOR = adjusted odds ratio | | | | | | | | | |

**Sensitivity Analysis**

**1.1**

Participants varied in the number of suicidal events they experienced. Most participants with suicidal events experienced one or two events, with one participant endorsing six events. We conducted supplementary analyses, which randomly sampled two of the six events for that participant to be included in analyses. Removing data increased the p-value; however, the effect size remained consistent with original findings of weekday time-in-bed predicting next-week suicidal events (OR [95% CI]=1.91[0.89, 3.87], *p*=.098).

**1.2**

To assess model robustness, we performed several techniques. First, we bootstrapped a Bayesian mixed model with 100 bootstrap replicates, which yielded similar estimates to our original solution, showing that a 1 SD increase in weekday time-in-bed (TIB), relative to one’s typical weekday TIB, related to a 2-folds greater odds of a next-week suicidal event (OR [95% CI]=2.00[1.35, 2.76], **Figure S1A**). Next, to evaluate the stability of this effect, we computed the median posterior probability that *β*>0 is ≥0.95. We found that the posterior median for effect of in weekday TIB relative to one’s typical weekday TIB on suicide events is 0.98 with a 95% CI between 0.74 and 0.99 (**Figure S1B**), indicating there is evidence for this effect. Finally, given bootstrapping could overrepresent non-suicide events, we supplemented our analyses with univariate (single predictor) analyses, which showed that 1 SD increase in weekly TIB, relative to one’s typical weekly TIB, related to a near 2-folds greater odds of a next-week suicidal event (OR [95% CI]=1.80[1.03, 3.07], *p*=.039). As in our primary multivariate analysis, this was driven by weekday (i.e., school week) TIB (OR [95% CI]=2.10[1.16, 3.65], *p*=.013).

**Figure S3.**

| **Table S3. Associations between Time-in-Bed and Clinically Significant Suicidal Ideation** | | | | | | | | | |
| --- | --- | --- | --- | --- | --- | --- | --- | --- | --- |
|  | **Next-Week SI** | | | **Next-Week SI** | | | **Next-Week SI** | | |
| *Predictors* | *aOR* | *95% CI* | *p* | *aOR* | *95% CI* | *p* | *aOR* | *95% CI* | *p* |
| Whole-Week TIB SD (pmean) | 1.00 | 0.74 – 1.36 | 0.99 |  |  |  |  |  |  |
| Whole-Week TIB Mean (pmean) | 0.89 | 0.66 – 1.21 | 0.48 |  |  |  |  |  |  |
| Baseline Suicidal Ideation Severity | **3.02** | **1.92 – 4.72** | **<0.001** | **3.01** | **1.90 – 4.65** | **<0.001** | **3.46** | **1.92 – 6.01** | **<0.001** |
| Weekday TIB Mean (pmean) |  |  |  | 0.85 | 0.63 – 1.17 | 0.33 |  |  |  |
| Weekday TIB SD (pmean) |  |  |  | 1.05 | 0.77 – 1.42 | 0.79 |  |  |  |
| Weekend TIB Mean (pmean) |  |  |  |  |  |  | 1.02 | 0.70 – 1.47 | 0.93 |
| Weekend TIB SD (pmean) |  |  |  |  |  |  | 1.26 | 0.87 – 1.78 | 0.22 |
| *Random Effects* |  |  |  |  |  |  |  |  |  |
| σ^2^ | 3.29 |  |  | 3.29 |  |  | 3.29 |  |  |
| τ_00_ | 2.55 _ID_ | |  | 2.55 _ID_ |  |  | 4.10 _ID_ |  |  |
| ICC | 0.44 |  |  | 0.44 |  |  | 0.55 |  |  |
| N | 115 _ID_ |  |  | 114 _ID_ |  |  | 102 _ID_ |  |  |
| Observations | 1341 |  |  | 1307 |  |  | 1013 |  |  |
| Marginal R^2^ / Conditional R^2^ | 0.173 / 0.535 |  |  | 0.172 / 0.534 |  |  | 0.173 / 0.632 |  |  |
| *Note.* TIB=Time-in-Bed; SD = standard deviation; pmean = person-centered; aOR = adjusted odds ratio | | | | | | | | | |

**References**

1.	Madigan S, Korczak DJ, Vaillancourt T, et al. Comparison of paediatric emergency department visits for attempted suicide, self-harm, and suicidal ideation before and during the COVID-19 pandemic: a systematic review and meta-analysis. *Lancet Psychiatry*. 2023;10(5):342-351. doi:10.1016/S2215-0366(23)00036-6

2.	Rocha S, Fuligni A. The impact of the COVID-19 pandemic on adolescent sleep behavior. *Curr Opin Psychol*. 2023;52:101648. doi:10.1016/j.copsyc.2023.101648
